# Shifting season of fire and its interaction with fire severity: Impacts on reproductive effort in resprouting plants

**DOI:** 10.1002/ece3.8717

**Published:** 2022-03-18

**Authors:** Alexandria M. Thomsen, Mark K. J. Ooi

**Affiliations:** ^1^ 7800 School of Biological, Earth and Environmental Sciences Centre for Ecosystem Science University of New South Wales Sydney New South Wales Australia; ^2^ NSW Bushfire Risk Management Research Hub Sydney New South Wales Australia

**Keywords:** climate change, fire regime, fire season, post‐fire flowering, prescribed burn, resprouting vigor

## Abstract

Fire regimes shape plant communities but are shifting with changing climate. More frequent fires of increasing intensity are burning across a broader range of seasons. Despite this, impacts that changes in fire season have on plant populations, or how they interact with other fire regime elements, are still relatively understudied. We asked (a) how does the season of fire affect plant vigor, including vegetative growth and flowering after a fire event, and (b) do different functional resprouting groups respond differently to the effects of season of fire? We sampled a total of 887 plants across 36 sites using a space‐for‐time design to assess resprouting vigor and reproductive output for five plant species. Sites represented either a spring or autumn burn, aged one to three years old. Season of fire had the clearest impacts on flowering in Lambertia formosa with a 152% increase in the number of plants flowering and a 45% increase in number of flowers per plant after autumn compared with spring fires. There were also season × severity interactions for total flowers produced for *Leptospermum polygalifolium* and *L. trinervium* with both species producing greater flowering in autumn, but only after lower severity fires. Severity of fire was a more important driver in vegetative growth than fire season. Season of fire impacts have previously been seen as synonymous with the effects of fire severity; however, we found that fire season and severity can have clear and independent, as well as interacting, impacts on post‐fire vegetative growth and reproductive response of resprouting species. Overall, we observed that there were positive effects of autumn fires on reproductive traits, while vegetative growth was positively related to fire severity and pre‐fire plant size.

## Graphical abstract

Shifting fire seasons can impact plant populations, but is still relatively understudied. We use five species to explore the impacts of changing fire season on vegetative growth and reproductive effort.

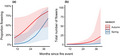

## 1 INTRODUCTION

Fire is a major factor shaping plant communities and their structure in many regions around the world (Bond & van Wilgen, 1996; McLauchlan et al., 2020; Whelan, 1995). As such, plant species have evolved to persist through fire regimes, broadly characterized by the season, intensity, and frequency of burns, typical of the region they occur in (Whelan, 1995). However, historical fire regimes are shifting, with changing climate and other anthropogenic factors producing more intense and more frequent burns (Enright et al., 2014; Flannigan et al., 2013; Le Breton et al., 2022; Pausas & Keeley, 2021). Seasonality of fire is also being altered, with projected decreases in precipitation and increased temperatures likely to produce longer fire seasons and increased fire likelihood out of historical peak seasons (Balch et al., 2017; Bradstock, 2010; Enright et al., 2014; Ooi, 2019).

In fire‐prone areas, plant species are categorized into two main post‐fire regeneration groups: resprouters and post‐fire seeders (Pausas & Keeley, 2014). Obligate seeders are those species where individuals do not resprout, but are dependent on fire‐cued germination or seed release for regeneration (Clarke et al., 2015). Resprouters are less dependent on seeds than obligate seeders for recovery after fire (Whelan, 1995). Instead, a proportion of resprouting plants survive fire, relying on growing new shoots from dormant buds (Ott et al., 2019), either protected under bark or from below‐ground structures (such as lignotubers or rhizomes), to resprout after a fire (Clarke et al., 2013). Protection of these vital plant tissues from the heat of the fire is a type of resistance strategy (Pausas, 2019). Epicormic (aerial) resprouters retain their main trunk after a fire and resprout from protected dormant buds under thick bark. Lignotuberous species are a type of basal resprouter that use the soil to insulate axillary buds, which can initiate resprouting of multiple stems after fire, even when some or all the above‐ground biomass has been destroyed by fire. Some lignotuberous species are capable of basal and epicormic resprouting, and fire response usually depends on age of plant and severity of fire (Smith et al., 2018).

While fire effects on plants and their persistence is a well‐studied subject, most attention has been given to the effects of fire frequency and, to a slightly lesser extent, intensity (Bellingham & Sparrow, 2000; Fairman et al., 2015). Comparatively, little research effort has been focused on seasonality of fire, especially on post‐fire flowering (Miller et al., 2019). Many studies that have investigated fire season shifts have tended to assume that any impacts on plant response is due to decreases in fire intensity, especially if out‐of‐season fires occur in the cooler months (Miller et al., 2019). However, it is likely that there is a number of interacting elements at play, including changing intensity, climatic effects, and distinct species phenology (Miller et al., 2020; Nolan et al., 2021). Recent reviews (Miller et al., 2019; Tangney et al., 2020) have shown that there is evidence for altered fire seasonality having considerable impact on post‐fire plant response, and highlighted the need for further studies. With fire regimes already shifting, there is an imperative to assess fire season impacts on plants in order to gain a deeper understanding of persistence and potential threats they face (Enright et al., 2014).

Obligate seeders are particularly susceptible to population decline under high fire frequency (Bradstock et al., 1997). In fact, fire frequency effects on obligate seeders are the basis of many current fire management guidelines (Bradstock et al., 1997; Foster et al., 2018). In contrast, resprouters are thought to be more resilient to increased fire frequency but perhaps more at risk from changes to fire intensity (Bellingham & Sparrow, 2000; Bennett et al., 2016). However, the timing of disturbance is also likely to affect resprouting ability. A plant's life cycle is strongly influenced by season and climate. Species will adapt to their environment by accumulating carbohydrates, sprouting new leaves, and flowering and fruiting at specific times of the year (Alvarado et al., 2014). Plants photosynthesize to obtain carbon, a small amount of which will be used to create nonstructural carbohydrates (NSC). These carbohydrates can be stored and utilized for vegetative growth and reproduction in the growing season, and resprouting after disturbances (Cruz et al., 2002; Martínez‐Vilalta et al., 2016). In temperate regions, plants generally increase their starch (an NSC) levels in winter and deplete them over the growing season (spring) (Martínez‐Vilalta et al., 2016). Utilizing NSCs, resprouters have the capacity to reshoot from protected buds, or underground storage structures after fire (Bradstock & Myerscough, 1988). In a study of the resprouting shrub *Erica australis* from fire‐prone Mediterranean Spain, Cruz and Moreno (2001) found that lignotuber total NSC concentrations was highest just before the growing period (spring) and decreased as the species entered its reproductive phase in summer. This seasonal fluctuation of resources means that the timing of a fire event could have considerable effects on the rate of vigor of resprouting and recovery (Klimešová & Klimeš, 2007). For example, plants that are burnt later in the growing season would have less time to grow and recover carbohydrate reserves before the next dormant season compared to if burned earlier in the season (de Groot & Wein, 2004; Schutz et al., 2009).

Fire can be very important for stimulating resprouting plants to flower, and in some regions post‐fire flowering is a dominant feature (Zirondi et al., 2021). Season of fire is likely to play a significant role too, yet studies are rare (Pyke, 2017). In some Australian examples, very sparse flowering has been recorded occurring during fire‐free intervals for *Blandfordia nobilis* (Johnson et al., 1994), *Xanthorrhoea fulva* (Taylor et al., 1998), *Telopea speciosissima* (Denham & Auld, 2002), and *Stirlingia latifolia* (Bowen & Pate, 2004), all of which are classified as pyrogenic resprouters. For some species, studies have shown that season of fire can play a part in increasing the proportion of individuals that flower. For example, Bowen and Pate (2004) found in *Stirlingia latifolia* that flowering occurred in 92% of plants burnt in summer/autumn compared with 73% of plants burnt in spring and less than 3% of individuals in areas that had not been burnt within 2 years.

Studying plant species grouped by their different mechanisms of resprouting response, including basal or epicormic sprouting and post‐fire flowering, provides a basis for interpreting findings more broadly. Studies investigating fire season response across different resprouting plant functional types have rarely, if ever, previously been made (Miller et al., 2019). Basal resprouting is an ancient trait that has arisen in several lineages (Keeley & Brennan, 2012) and is a common response to a range of natural events such as drought, frost, herbivory, storm damage, and fire (Bradshaw et al., 2011). Epicormic resprouting is globally less common (Pausas & Keeley, 2017), and it has been hypothesized that it arose in the Myrtaceae family in the Cenozoic (~60–62 Mya) and has evolved in flammable sclerophyll biomes (Crisp et al., 2011). There is therefore support that epicormic resprouting is an adaptation to fire, whereas basal resprouting may have evolved under several selective forces over time. These different origins may determine a species’ ability to cope with fire regime changes.

In this study, we set out to investigate the effects of fire seasonality on resprouting and reproductive responses of species in temperate dry sclerophyll forests of southeastern Australia, a region with an aseasonal rainfall climate. These plant communities have their woody shrub layer dominated by resprouter and obligate seeder functional groups (Clarke et al., 2015). Notably, we aimed to control for the effects of fire seasonality from fire intensity (correlated to fire severity estimates, see Keeley, 2009) to highlight the impacts that shifting fire timing alone may have. Wildfires in these plant communities historically occur from spring to summer, generally burn at a medium to high intensity (resulting in 50–100% canopy consumption) (Bradstock, 2010), and occur every 10 to 50 years (Enright & Thomas, 2008; Gill & Moore, 1990), whereas prescribed burns are of a much lower intensity and occur in the cooler seasons of mid‐autumn to mid‐spring (McLoughlin, 1998). Wildfires across Australia are predominantly ignited by lightning strikes in summer months when there is a sufficient fuel load (Enright & Thomas, 2008); however anthropogenic factors, including climate change, are also shifting the season in which fires occur. As such, anthropogenic factors are lengthening the fire season and changing fire seasonality. More specifically, the objectives of this study will address the following questions:

1. How does the season of fire affect plant vigor, including vegetative growth and flowering, after a fire event?
2. Do the different functional resprouting groups (i.e., basal resprouting species, or mixed basal/epicormic resprouting species) respond differently to the effects of season of fire?

## 2 MATERIALS AND METHODS

### 2.1 Study species

**FIGURE 1:**
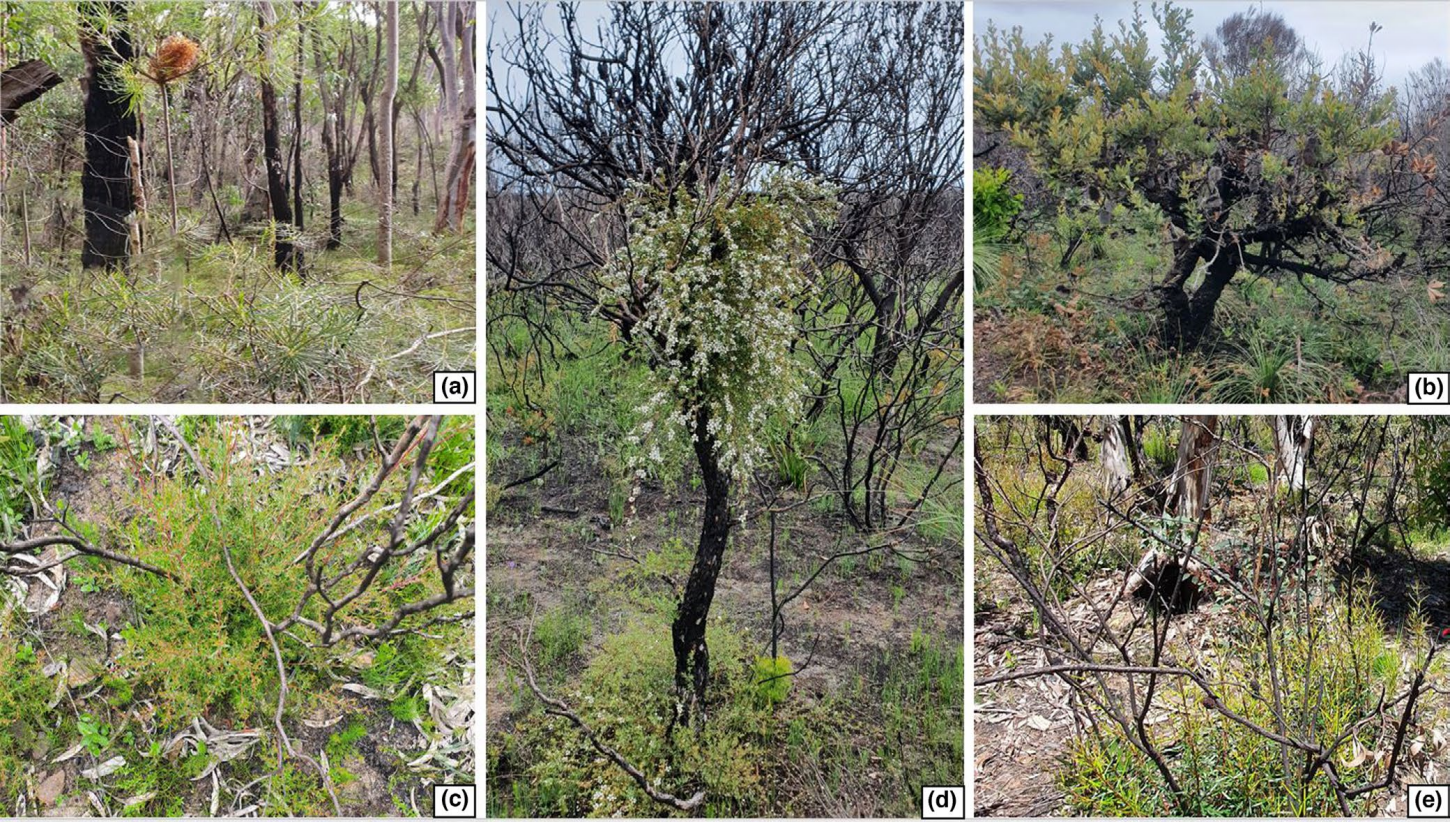
Photos of the five study species: (a) basal resprouter *Banksia spinulosa*, (b) epicormic resprouter *Banksia serrata*, (c) basal resprouter *Leptospermum polygalifolium*, (d) epicormic resprouter *Leptospermum trinervium*, and (e) basal resprouter *Lambertia formosa*

Five species from the Proteaceae and Myrtaceae families were examined to test the effects of seasonality of fire (Figure 1, Table 1). These families both have lignotuberous species that display traits of both epicormic and basal resprouting and are two of those most common plant families in the region. Species were chosen if they produced a resprouting response from a lignotuber after fire and had a wide enough distribution with reasonable abundance over replicated study sites with different seasons of burn. A sample of species from different resprouting functional groups, including basal and epicormic resprouters, were included. Note that all epicormic species chosen can both epicormically and basally resprout and all species are common in Sydney dry sclerophyll forests (Benson & Howell, 1994). Out of the five species, *L*. *formosa* is the only species dependent on fire being a facultative pyrogenic flowerer, and the remaining four species are able to flower in the absence of fire.

**TABLE 1:** Information for each of the species used in the study. Number of individuals sampled are data for all sites combined

Family	Species	Growth form	Resprout organ	Resprouter type	Flowering period	Number of individuals sampled
Proteaceae	*Banksia serrata*	Shrub or tree up to 10 m	Lignotuber	Basal × epicormic	Jan–June	267
Proteaceae	*Banksia spinulosa*	Multi‐stemmed shrub up to 3 m	Lignotuber	Basal	April–Aug	103
Proteaceae	*Lambertia formosa*	Shrub up to 2 m	Lignotuber	Basal	All year	163
Myrtaceae	*Leptospermum polygalifolium*	Shrub up to 4 m	Lignotuber	Basal	Aug–Jan	88
Myrtaceae	*Leptospermum trinervium*	Shrub or tree up to 5 m	Lignotuber	Basal x epicormic	Spring	266

### 2.2 Study region and site selection

All sites were within National Parks (NP) in the Sydney region of New South Wales (NSW), Australia, located on sandstone soils and consisted of dry sclerophyll forest and/or woodland. The most northern site was situated in Berowra Valley NP (−33.634084, 151.111876) and the most southern site was in Dharawal NP (−34.161309, 150.830847). Other National Parks included were Lane Cove, Garigal, Ku‐Ring‐Gai Chase, Royal, and Heathcote. The climate is temperate with aseasonal rainfall, and no prominent dry season (humid subtropical (Cfa) using the Köppen Climate Classification). Average annual rainfall for the years 2016–2019 ranged from 927 mm to 1093 mm. The average monthly temperature (max/min) for summer is 26/18°C, and that for winter is 17/8°C (Terrey Hills AWS station, Australian Government Bureau of Meteorology, 2019). Historically, dry sclerophyll forests of the region experience wildfire every 10–50 years during late spring‐summer. Where possible, all sites selected had similar fire history, where the penultimate fire was 15–20 years ago.

We used a space‐for‐time design to assess resprouting vigor and reproductive output of the five study species in response to fires in different seasons. Fire history records from the NSW Department of Planning, Industry and Environment were used to determine potential suitable areas in the study region. All sampling was carried out in 2019 in areas that had been burnt in either 2016, 2017, or 2018. At least five spring and five autumn‐burnt sites from each year (2016–2018) were identified. The time period of up to 3 years post fire was chosen, as many resprouting plants are likely to flower within this period (Burrows et al., 2008); however, one species (*Banksia spinulosa*) needed to be removed from the reproduction analysis due to limited flowering in this time frame. Fire severity at each site was measured using minimum burn tip diameter of burnt shrubs. This method is often used for post hoc assessment of fire intensity (Hammill & Bradstock, 2006; Moreno & Oechel, 1989) and found to correlate with fire severity (*sensu* Keeley, 2009) (Whight & Bradstock, 1999), as well as plant ecological processes including resprouting (Knox & Clarke, 2016; Pérez & Moreno, 1998). To assess variation in severity of fire between sites, five branch tips at chest height were measured on, where possible, 10 individual plants at each site (predominately *L*. *trinervium*) using digital calipers. The larger the minimum branch tip diameter remaining, the greater the amount of biomass consumed, indicating greater heat release (Moreno & Oechel, 1989). The severity score therefore increases with thickness of the remaining branch tip, with low severity burns consuming no to little branch biomass resulting in small scores, to high severity burns consuming through thicker branches and resulting in larger scores. The average of the total tip measurements was calculated to give a severity score for each site (Appendix S1).

### 2.3 Sampling design

At each site, a random location was established and a 30 m transect laid out. All target species within a 2 m distance from the transect were assessed for resprout growth and reproductive output between May and July 2019. A total of 887 plants were measured across 36 sites. Where possible, at least 10 individuals of each species per site were used, with at least a total of 88 individuals sampled per species (Table 1). The variables measured included (i) vegetative growth (number of resprouts × length of resprouts), either by epicormic shoots or basal shoots from a lignotuber, and reproductive response (ii) proportion of individuals flowering and (iii) number of flowers and fruits produced. Number of inflorescences were counted for *Banksia serrata* and *L*. *formosa* but are still referred to as number of flowers in results. Size characteristics of each individual plant pre‐fire were estimated by measuring height and diameter (at 1 m high) of the thickest stem or trunk, and width of base of growth. Diameter of stem was found to be the most consistently available measurement to infer size/age of plant before fire across all the study species, and so was used in the analysis. For the study species with basal and epicormic resprouting, *B*. *serrata* and *L*. *trinervium*, data were analyzed for each response separately, to test for the effects of seasonality on different resprouting mechanisms.

### 2.4 Statistical analysis

All analyses were carried out in R Studio version 1.3.1093 (RStudio Team, 2020). For the vegetative growth response variable, data were analyzed for each species separately using a Linear Mixed Model (LMM) in the *lme4* package (Bates et al., 2015). Season of fire, age of regrowth (time since fire event), diameter, and severity of burn were included in the model as fixed factors, with season x age of regrowth and season x severity interaction terms also included in the global model. Site was included as a random factor. Severity was analyzed as a continuous variable, ranging from mean tip diameters of 0.4 to 2.68 mm. We assessed whether residuals from each global model was normally distributed using histogram plots and used log transformation prior to analysis for any that did not meet this assumption. For the number of flowers produced, we analyzed both the total flowers produced per site across all plants observed, and the number of flowers produced only for individuals that flowered. The same model terms were used as for vegetative growth but in a negative binomial Generalized LMM (GLMM) (nbinom2 in the *glmmTMB* package, Brooks et al., 2017), due to overdispersion when analyzed with a Poisson model. Note that for *L*. *polygalifolium*, there were too few data for the full model to converge, so we ran a reduced model with no interactions and tested for significance using the analysis of variance (ANOVA) function on this reduced model. For *L*. *polygalifolium*, age was also removed as a factor due to limited flowering in recently burnt sites. To analyze the proportion of plants flowering, we used a GLMM with a binomial error distribution and included age of regrowth, season, severity, and an age x season interaction term in the models, with site again included as a random factor. For the two epicormic species (*B*. *serrata and L*. *trinervium*), we further tested the likelihood of flowering from resprout position (basal shoots or epicormic shoots) using a GLMM with a binomial error distribution with the same terms of the global vegetative growth model. For all dependent variables, model selection was used to determine the best‐fitting model and reveal the strongest predictors (Johnson & Omland, 2004). Fitted models were compared with Akaike's information criteria (AICc) and the model with the strongest empirical support (i.e., the lowest AICc) was used. Significance of factors were estimated for the best‐fitting models using the ANOVA function in the R package *car* (Fox, 2019).

## 3 RESULTS

### 3.1 Proportion of individuals flowering

**FIGURE 2:**
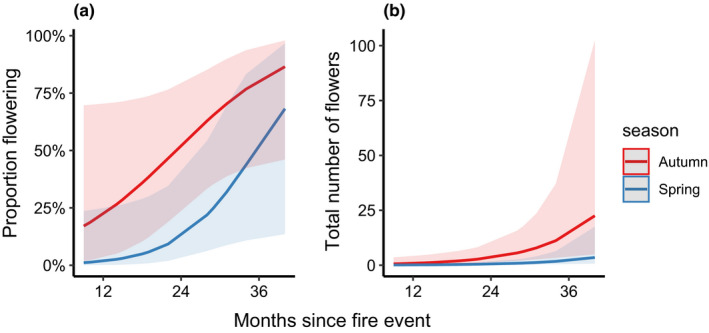
Predicted values for reproductive effort: (a) mean proportion flowering and (b) mean total number of flowers against time since fire event in *Lambertia formosa* after both autumn and spring fires

All species showed evidence of flowering during the study period; however, *B*. *spinulosa* only flowered at one site that was burnt in a spring 2016 fire and so was removed from the seasonal fire comparisons. Differences in flowering, when analyzed across all years, were found between fire season for *L*. *formosa* (df = 1, χ^2^ = 4.046, *p* = .044). On average, there was a 152% increase of flowering plants after autumn burns compared to spring burns (Figure 2a). Plants also took longer to flower post spring burns with a mean of 10% plants flowering compared to 83% after autumn burns, 2 years post fire. No significant difference was found for the other three study species.

### 3.2 Number of flowers

For the total number of flowers produced (a measure of reproduction at the site level) and number of flowers per flowering individual (a measure of reproductive effort per plant), there were main effects of both season and severity, as well as season x severity interactions. Size of the parent plant (diameter) was also included as a predictor in the best‐fitting models for three species (Table 2).

**TABLE 2:** Model selection outcomes and factor significance for number of flowers produced at both the site level and for individual plants that flowered

Species	Fire Response	Model factors									
		Number of flowers (Total)					Number of flowers (per flowering individual)				
		Age	Diameter	Season	Severity	Season x severity	Age	Diameter	Season	Severity	Season x severity
*Banksia serrata*	Epicormic									**	
*Lambertia formosa*	Basal	***		*					*	*	
*Leptospermum polygalifolium*	Basal							*	**	*	
*Leptospermum trinervium*	Basal						***	**			
*Leptospermum trinervium*	Epicormic					***					**

Note

Factors included are time since fire event (age), diameter of trunk of the pre‐fire plant, season of fire event, severity of fire, and a season x severity interaction term. Gray‐highlighted cells show factors that were included in best‐fitting models (based on lowest AICc scores) for different dependent variables for each species. Asterisks denote where factors were significant predictors (*p < .05, **p < .01, ***p < .001).

**FIGURE 3:**
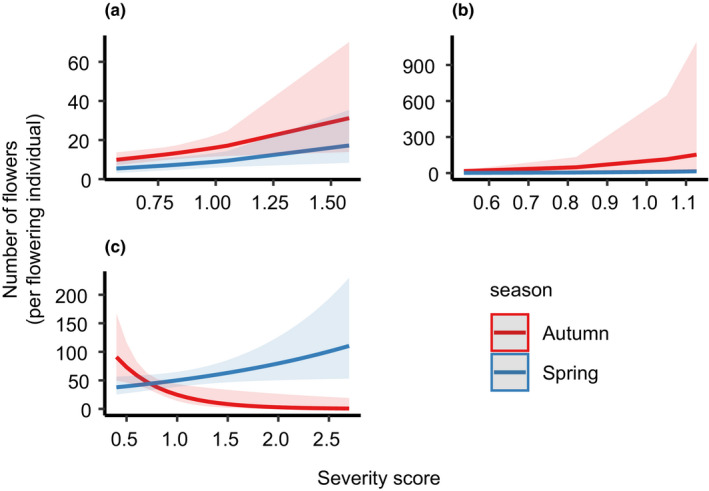
Predicted mean number of flowers (per flowering individual) in spring and autumn against fire severity score for (a) *Lambertia formosa*, (b) *Leptospermum polygalifolium*, and (c) epicormically resprouting *Leptospermum trinervium*

Season and age of regrowth were the best predictors for total number of flowers produced by *L*. *formosa* (df = 1, χ^2^ = 4.845, *p* = .028) (Figure 2b). On average, a 45% increase of number of flowers was seen after autumn fires compared to spring burns. There were also main effects of season for the number of flowers produced per flowering individual for *L*. *polygalifolium* (df = 1, χ^2^ = 7.488, *p* = .006) (Figure 3b) and, again, *L*. *formosa* (df = 1, χ^2^ = 2.944, *p* = .086) (Figure 3a). Both species produced more flowers in autumn compared to spring. There was a strong interaction between season and severity for total flowers produced for epicormically resprouting *L*. *trinervium* (df = 1, χ^2^ = 13.227, *p* < .001). There was also a strong interaction between season and severity present in the best‐fitting model for *L*. *polygalifolium* for total flowers (df = 1, χ^2^ = 3.004, *p* = .083) (Table 2). Greater flowering occurred at autumn‐burnt sites, but only after lower severity fires. This was reversed for higher severity fires, with more flowers produced in spring. The same pattern was found for *L*. *trinervium* for flowers per reproductive individual (df = 1, χ^2^ = 8.573, *p* = .003) (Figure 3c).

**FIGURE 4:**
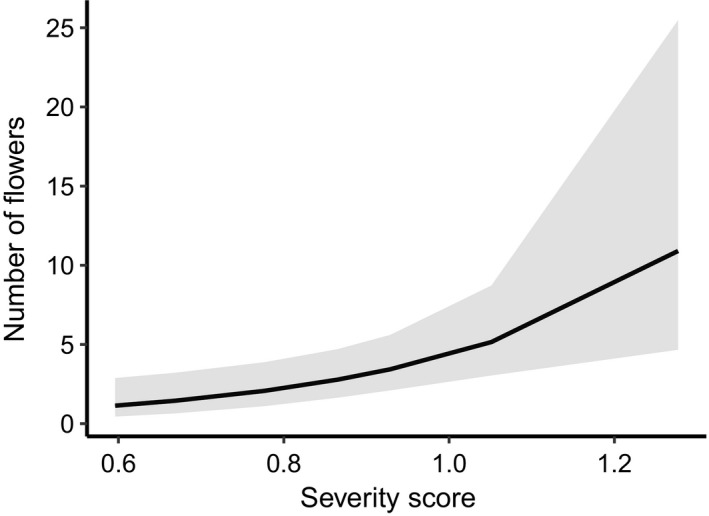
Predicted mean number of flowers against fire severity score (measured as stem tip diameter of remaining branches) for *Banksia serrata*

There was a main effect of severity for the number of flowers produced per flowering individual for *B*. *serrata* (df = 1, χ^2^ = 8.629, *p* = .003) (Figure 4), *L*. *formosa* (df = 1, χ^2^ = 5.389, *p* = .020) (Figure 3a), and *L*. *polygalifolium* (df = 1, χ^2^ = 5.878, *p* = .015) (Figure 3b). Flower numbers increased per plant with increasing fire severity. However, there was no main effect of severity for total flower production for any species.

### 3.3 Likelihood of flowering with resprout response

**FIGURE 5:**
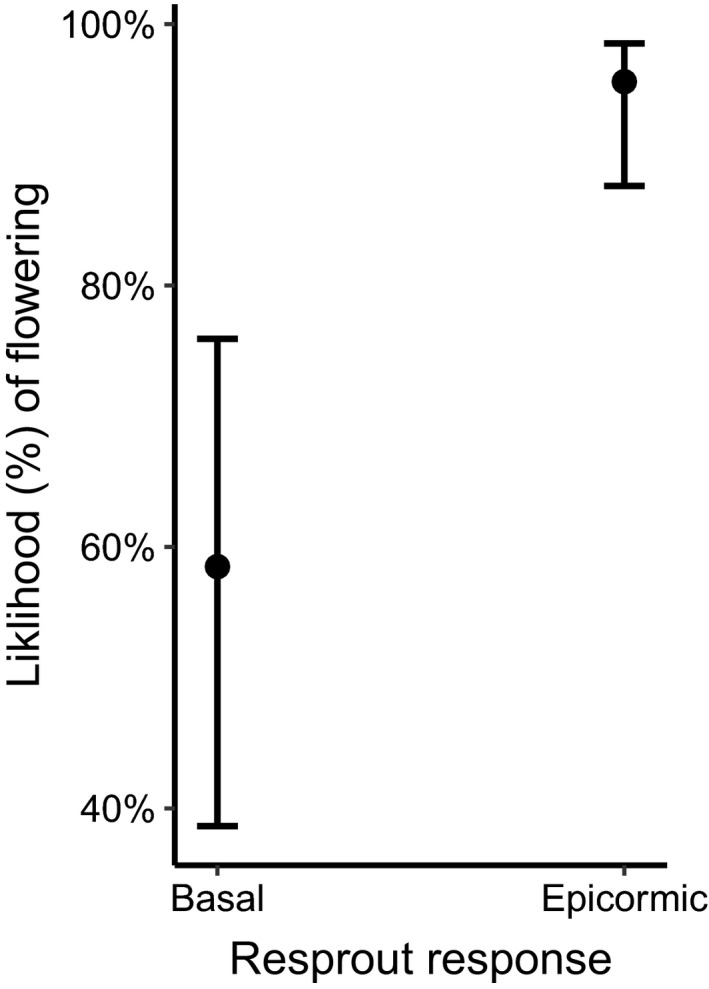
Predicted likelihood of flowering (%) in *Leptospermum trinervium* in individuals that resprouted via basal resprouts or epicormic resprouts

For the two mixed‐response species (*B*. *serrata* and *L*. *trinervium*), we tested for main drivers of likelihood of flowering. For *L*. *trinervium*, resprout response type and age of resprouts were found to be the best predictors of likelihood of flowering (resprout response; df = 1, χ^2^ = 27.136, *p* < .0001, age; df = 1, χ^2^ = 2.377, *p* = .1232). Individuals of *L*. *trinervium* were on average 53% more likely to flower on epicormic shoots compared to basal shoots (Figure 5). Due to the timeframe over which *B*. *serrata* was surveyed in, we saw minimal flowering and could not statistically analyze drivers of likelihood of flowering. However, within the 3 years post fire, we observed zero flowering in plants that had resprouted basally, and a mean of 8% flowering on epicormically resprouted individuals.

### 3.4 Vegetative growth responses

**FIGURE 6:**
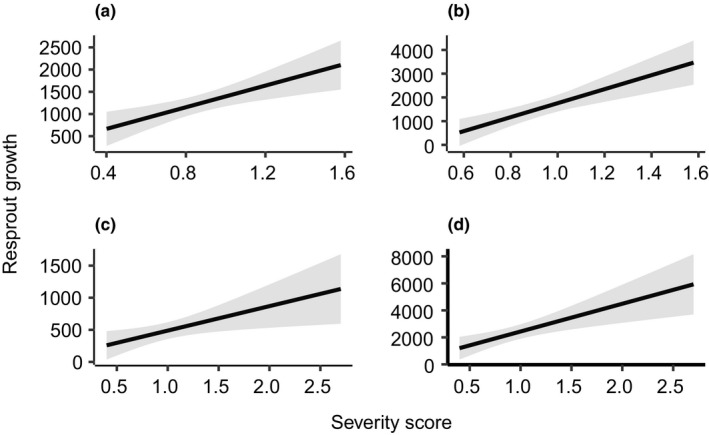
Predicted values of mean resprout growth (length of longest resprout × number of resprouts) in relation to fire severity score for (a) *Lambertia formosa*, (b) *Banksia spinulosa*, (c) basally resprouting *Leptospermum trinervium*, and (d) epicormically resprouting *Leptospermum trinervium*

Vegetative growth was strongly positively related to size of the parent plant (diameter) for all study species, while fire severity was also a significant driver of growth (Table 3). Positive effects of fire severity were found for *B*. *spinulosa* (df = 1, χ^2^ = 18.281, *p* < .001), *L*. *formosa* (df = 1, χ^2^ = 10.678, *p* = .001), and *L*. *trinervium* (both basal df = 1, χ^2^ = 11.601, *p* < .001 and epicormic df = 1, χ^2^ = 18.810, *p* < .001) (Figure 6). Age of regrowth was the only determining factor for vegetative growth for *L*. *formosa* and *B*. *serrata* (both basal and epicormically resprouters). Season of fire had no significant effect on vegetative regrowth (Table 3).

**TABLE 3:** Model selection outcomes and factor significance for vegetative growth and reproduction

Species	Fire Response	Vegetative growth			
		Age	Diameter	Season	Severity
*Banksia serrata*	Basal	*	*		
*Banksia serrata*	Epicormic	*	***		
*Banksia spinulosa*	Basal		***		***
*Lambertia formosa*	Basal		*		**
*Leptospermum polygalifolium*	Basal		*		
*Leptospermum trinervium*	Basal	**	*		***
*Leptospermum trinervium*	Epicormic	***			***

Note

Factors included time since fire event (age), diameter of trunk of the pre‐fire plant, season of fire event, and severity of fire. Gray‐highlighted cells show factors that were included in best‐fitting models for different dependent variables for each species. Asterisks denote where factors were significant predictors (*p < .05, **p < .01, ***p < .001).

## 4 DISCUSSION

The impact of season of fire on plant responses has lagged behind research on other fire regime elements in temperate ecosystems, especially in resprouting plants (Miller et al., 2019). Furthermore, season of fire impacts have previously been seen as synonymous with the effects of fire severity (Miller et al., 2020). In our study, we found that fire season and severity can have clear and independent impacts, as well as interacting effects, on post‐fire vegetative growth and reproductive response of resprouting species. Overall, we observed that there were positive effects of autumn fires on proportion flowering and number of flowers, while vegetative growth was positively related to fire severity and pre‐fire plant size.

Of all our study species, the basal resprouter *L*. *formosa* displayed the clearest impacts by season of fire, with autumn burns consistently increasing the proportion of plants flowering and the number of flowers produced. The time taken for a high proportion of *L*. *formosa* plants to flower was also faster after autumn burns, compared to spring burns, effectively decreasing the secondary juvenile period. These results support other studies of pyrogenic flowering shrubs that show more vigorous flowering after autumn compared to spring burns in Mediterranean‐climate (Bowen & Pate, 2004) and aseasonal rainfall regions (Paroissien & Ooi, 2021). Our study region has an aseasonal rainfall climate, with no annual drought period, which highlights that post‐fire flowering variation in response to the season that fires occur is likely related to resourcing rather than seasonal stresses. Only 1%–5% of *L*. *formosa* flowers reach seed set each year (Pyke, 1982) and any further limitation of flowering imposed by season of burns could be detrimental to replenishment of stored seeds. These results highlight the need for further study of the facultative fire‐stimulated flowering group and fire season, particularly in regions where such species form a dominant part of the community (Lamont & Downes, 2011; Zirondi et al., 2021).

In addition to *L*. *formosa*, *L*. *polygalifolium* also produced more flowers per flowering individual after autumn burns. While intuitively this result could be explained by the fact that autumn‐burnt plants had a greater time to mature before the flowering season than their spring‐burnt counterparts, comparisons between older spring‐burnt plants (e.g., plants burnt in spring 2016 compared to those burnt in autumn 2017) still displayed an autumn fire advantage. This suggests that fire season has effects beyond this age difference. Other studies have also documented that autumn burns result in a greater reproductive effort, often attributing this to a longer period of recovery time between the fire event and flowering period (Bowen & Pate, 2004; Johnson et al., 1994; Nield et al., 2016). However, fires occurring just after the flowering season would allow maximum time to resprout and recover photosynthetic capacity before the next flowering period. Therefore, spring flowering plants that are burnt at the start of spring may more likely see delayed and reduced flowering, compared to those burnt 6 to 9 months before, in an autumn fire. Reduced flowering through altered fire season can reduce the number of seeds available for post‐fire recruitment, making them less competitive in the post‐fire environment (Lamont & Downes, 2011; Miller et al., 2019). This impact is then exacerbated in *L*. *formosa* as it would have to wait until the next fire event to regain peak flowering if it experienced a less favorable fire season.

Another hypothesis, that higher severity fire is needed to stimulate greater flowering particularly in some facultative pyrogenic species (Lamont & Downes, 2011; Nield et al., 2016; Taylor et al., 1998), was supported by our results. We found that fire severity had significant independent effects on the numbers of flowers produced per reproductive plant for *L*. *formosa*, *B*. *serrata*, and *L*. *polygalifolium*. This is likely to be driven, in part, by the positive relationship between fire severity and vegetative growth for three species in our study, with larger resprout growth providing a greater platform for flowering. However, the relationship between severity and resprouting vigor was not found for all study species, reflecting similar mixed results from other studies of Australian shrubs (Knox & Clarke, 2011), and suggesting an interplay between mechanisms of bud insulation (Pausas & Keeley, 2017) and activation from bud dormancy (Ott et al., 2019). For *B*. *serrata*, there was a positive relationship between resprouting vigor and trunk diameter and a difference between the basal and epicormics response (see Figure S2). However, the lack of impact of fire severity on resprouting vigor in our study was possibly due to the relatively low fire severities produced by prescribed burns combined with this species size and level of bud protection. *B*. *serrata* grows to 10 m and has thicker “corky” bark, which is likely to better insulate epicormic buds and reduce flammability (Pausas & Keeley, 2017). The low fire severities, at least relative to those produced during wildfires, may mean that buds were not activated (see Ott et al., 2019), due to the scorch heights being low and defoliation being limited. Conversely, the smaller size and thinner bark of the other study species promoted a positive resprouting vigor response within the bounds of the severity reached.

The effects of fire severity on resprouting vigor are not clear, with primarily negative but also positive relationships found across multiple studies. Generally, negative effects of increasing fire severity are attributed to plant and bud mortality, especially at high or extreme fire intensities (Fernández et al., 2013; Lloret & López‐Soria, 1993; Moreno & Oechel, 1991). However, positive effects are also found for high‐intensity fires (Knox & Clarke, 2011; Strydom et al., 2020). We assessed the role that fire severity can play across different seasons of cool prescribed burns, not including the much higher severities that cause bud mortality. Although, season of fire can influence intensity through changes in weather, fuel load, and flammability of plant material (Whelan, 1995), the effects of severity as a driver of resprouting and flowering within the context of our study highlight that impacts are likely to be more complex than heat‐induced mortality. Furthermore, we found an interaction between fire season and severity for the total number of flowers produced for *L*. *trinervium*, with greater flowering in autumn at lower severities, but the opposite after higher severity fires. This differed to the result for vegetative resprouting vigor for this species, which showed a uniform increase with severity. Seasonally‐driven resource allocation is one possible variable driving the seasonal interaction with fire severity for *L*. *trinervium* flowering response. Bud banks in many fire‐prone species are dormant until fire activates them (Ott et al., 2019), and greater severity within the range of our study appears to increase the proportion of buds activated. However, subsequent growth from activated buds is supported by available plant resources (Hiers et al., 2021; Ott et al., 2019), and carbohydrate reserves are likely to be lowest in autumn for *L*. *trinervium*, at the end of the active growth season. This suggests that sufficient resources were available for vegetative recovery after either burn season, but as bud activation increased after autumn burns (with fire severity), resources were too limiting for flowering.

It was difficult to identify clear differences in response to fire season by the two functional resprouting groups (basal or mixed epicormic/basal). Once separated into their component responses, mixed resprouters displayed different responses to different fire season for both vegetative and reproductive elements. However, while more species are required to thoroughly compare functional groups, it is interesting that the two basal resprouters displayed the clearest impacts of season of burn, particularly for reproductive response. This may be related to the fact that epicormic resprouters, assumed to have evolved in response to fire (Crisp et al., 2011), may also be more resilient to fire regime changes, as seen from studies investigating increases in fire intensity and frequency (Collins et al., 2019). Furthermore, when comparing resprouting and reproductive efforts between resprout response within species, both mixed‐response species (*B*. *serrata* and *L*. *trinervium*) showed increased vegetative and flowering efforts when resprouting epicormically than basally. Severity of fire has been shown to correlate with resprout response, with severe fires leading to mortality of epicormic buds and resprouting basally only. Our research did not experience this mortality threshold, and the differences in resprouting response are most likely to be correlated with age (trunk diameter) (see Appendices S1 and S2), again highlighting the importance of maintain a diverse age structure in populations.

One of our clearest findings, that flowering output was driven by fire season, has implications for implementing burns, particularly if they are focused on species management. For example, the consistent relationship between season of fire and flowering of *L*. *formosa*, a species strongly dependent on fire for significant reproduction, suggests that season of burn should be considered when managing facultative pyrogenic flowerers. We would recommend that autumn burns should be preferred over spring burns in this region when this plant functional group is a conservation focus, a finding supported by studies of other pyrogenic flowering species (e.g., Paroissien & Ooi, 2021). However, further research needs to be conducted to see if the differences found have any long‐term impacts or how broadly such impacts are across multiple functional groups (Miller et al., 2019; Nolan et al., 2021). Use of prescribed burns and climate change are both altering fire seasonality and, as the results of this study show, can have independent impacts or interact with fire severity and potentially other fire regime elements. Understanding these impacts and interactions is essential for more informed fire management into the future.

## CONFLICT OF INTEREST

The authors declare that the research was conducted in the absence of any commercial or financial relationships that could be construed as a potential conflict of interest.

## AUTHOR CONTRIBUTIONS

**Alexandria M. Thomsen:** Conceptualization (equal); Data curation (lead); Formal analysis (lead); Methodology (equal); Writing – original draft (lead); Writing – review & editing (equal). **Mark K. J. Ooi:** Conceptualization (equal); Formal analysis (supporting); Funding acquisition (lead); Methodology (equal); Supervision (lead); Writing – original draft (supporting); Writing – review & editing (equal).

### OPEN RESEARCH BADGES

This article has earned an Open Data Badge for making publicly available the digitally‐shareable data necessary to reproduce the reported results. The data is available at https://doi.org/10.6084/m9.figshare.15079248.v2.

## Supporting information

Appendix S1

Fig S2

Appendix S2

## Data Availability

The datasets related to this study are available at https://doi.org/10.6084/m9.figshare.15079248.v2 (Thomsen & Ooi, 2021).

## References

[ece38717-bib-0001] Alvarado, S. T. , Buisson, E. , Rabarison, H. , Rajeriarison, C. , Birkinshaw, C. , Lowry, P. P. II , & Morellato, L. P. (2014). Fire and the reproductive phenology of endangered Madagascar sclerophyllous tapia woodlands. South African Journal of Botany, 94, 79–87. 10.1016/j.sajb.2014.06.001

[ece38717-bib-0002] Australian Government Bureau of Meteorology . (2019). Climate Data Online. http://www.bom.gov.au/climate/data

[ece38717-bib-0003] Balch, J. K. , Bradley, B. A. , Abatzoglou, J. T. , Nagy, R. C. , Fusco, E. J. , & Mahood, A. L. (2017). Human‐started wildfires expand the fire niche across the United States. Proceedings of the National Academy of Sciences, 114(11), 2946–2951. 10.1073/pnas.1617394114 PMC5358354 28242690

[ece38717-bib-0004] Bates, D. , Mächler, M. , Bolker, B. , & Walker, S. (2015). Fitting linear mixed‐effects models using lme4. Journal of Statistical Software, 67(1), 1–48. 10.18637/jss.v067.i01

[ece38717-bib-0005] Bellingham, P. J. , & Sparrow, A. D. (2000). Resprouting as a life history strategy in woody plant communities. Oikos, 89, 409–416. 10.1034/j.1600-0706.2000.890224.x

[ece38717-bib-0006] Bennett, L. T. , Bruce, M. J. , MacHunter, J. , Kohout, M. , Tanase, M. A. , & Aponte, C. (2016). Mortality and recruitment of fire‐tolerant eucalypts as influenced by wildfire severity and recent prescribed fire. Forest Ecology and Management, 380, 107–117. 10.1016/j.foreco.2016.08.047

[ece38717-bib-0007] Benson, D. , & Howell, J. (1994). The natural vegetation of the Sydney 1: 100 000 map sheet. Cunninghamia, 3(4), 677–787.

[ece38717-bib-0008] Bond, W. J. , & van Wilgen, B. W. (1996). Fire and plants. Chapman & Hall.

[ece38717-bib-0009] Bowen, B. J. , & Pate, J. S. (2004). Effect of season of burn on shoot recovery and post‐fire flowering performance in the resprouter *Stirlingia latifolia* R. Br. (proteaceae). Austral Ecology, 29(2), 145–155. 10.1111/j.1442-9993.2004.01332.x

[ece38717-bib-0010] Bradshaw, S. D. , Dixon, K. W. , Hopper, S. D. , Lambers, H. , & Turner, S. R. (2011). Little evidence for fire‐adapted plant traits in Mediterranean climate regions. Trends in Plant Science, 16, 69–76. 10.1016/j.tplants.2010.10.007 21095155

[ece38717-bib-0011] Bradstock, R. A. (2010). A biogeographic model of fire regimes in Australia: Current and future implications. Global Ecology and Biogeography, 19(2), 145–158. 10.1111/j.1466-8238.2009.00512.x

[ece38717-bib-0012] Bradstock, R. , & Myerscough, P. (1988). The survival and population response to frequent fires of two woody resprouters *Banksia serrata* and *Isopogon anemonifolius* . Australian Journal of Botany, 36, 415. 10.1071/BT9880415

[ece38717-bib-0013] Bradstock, R. A. , Tozer, M. G. , & Keith, D. A. (1997). Effects of high frequency fire on floristic composition and abundance in a fire‐prone heathland near Sydney. Australian Journal of Botany, 45, 641. 10.1071/BT96083

[ece38717-bib-0014] Brooks, M. E. , Kristensen, K. , Benthem, K. J. , Magnusson, A. , Berg, C. W. , Nielsen, A. , Skaug, H. J. , Mächler, M. , & Bolker, B. M. (2017). glmmTMB balances speed and flexibility among packages for zero‐inflated generalized linear mixed modeling. The R Journal, 9(2), 378–400. 10.32614/RJ-2017-066

[ece38717-bib-0015] Burrows, N. D. , Wardell‐Johnson, G. , & Ward, B. (2008). Post‐fire juvenile period of plants in south‐west Australia forests and implications for fire management. Journal of the Royal Society of Western Australia, 91, 163–174.

[ece38717-bib-0016] Clarke, P. J. , Lawes, M. J. , Midgley, J. J. , Lamont, B. B. , Ojeda, F. , Burrows, G. E. , Enright, N. J. , & Knox, K. J. E. (2013). Resprouting as a key functional trait: How buds, protection and resources drive persistence after fire. New Phytologist, 197, 19–35. 10.1111/nph.12001 23110592

[ece38717-bib-0017] Clarke, P. J. , Lawes, M. J. , Murphy, B. P. , Russell‐Smith, J. , Nano, C. E. M. , Bradstock, R. , Enright, N. J. , Fontaine, J. B. , Gosper, C. R. , Radford, I. , Midgley, J. J. , & Gunton, R. M. (2015). A synthesis of postfire recovery traits of woody plants in Australian ecosystems. Science of the Total Environment, 534, 31–42. 10.1016/j.scitotenv.2015.04.002 25887372

[ece38717-bib-0018] Collins, L. , Bennett, A. F. , Leonard, S. W. , & Penman, T. D. (2019). Wildfire refugia in forests: Severe fire weather and drought mute the influence of topography and fuel age. Global Change Biology, 25(11), 3829–3843. 10.1111/gcb.14735 31215102

[ece38717-bib-0019] Crisp, M. D. , Burrows, G. E. , Cook, L. G. , Thornhill, A. H. , & Bowman, D. M. (2011). Flammable biomes dominated by eucalypts originated at the Cretaceous‐Palaeogene boundary. Nature Communications, 2(1), 1–8. 10.1038/ncomms1191 21326225

[ece38717-bib-0020] Cruz, A. , & Moreno, J. M. (2001). Seasonal course of total non‐structural carbohydrates in the lignotuberous Mediterranean‐type shrub *Erica australis* . Oecologia, 128(3), 343–350. 10.1007/s004420100664 24549903

[ece38717-bib-0021] Cruz, A. , Pérez, B. , Quintana, J. R. , & Moreno, J. M. (2002). Resprouting in the Mediterranean‐type shrub *Erica australis* afffected by soil resource availability. Journal of Vegetation Science, 13(5), 641–650. 10.1111/j.1654-1103.2002.tb02092.x

[ece38717-bib-0022] de Groot, W. J. , & Wein, R. W. (2004). Effects of fire severity and season of burn on *Betula glandulosa* growth dynamics. International Journal of Wildland Fire, 13, 287–295. 10.1071/WF03048

[ece38717-bib-0023] Denham, A. J. , & Auld, T. D. (2002). Flowering, seed dispersal, seed predation and seedling recruitment in two pyrogenic flowering resprouters. Australian Journal of Botany, 50(5), 545–557. 10.1071/BT02009

[ece38717-bib-0024] Enright, N. J. , Fontaine, J. B. , Lamont, B. B. , Miller, B. P. , & Westcott, V. C. (2014). Resistance and resilience to changing climate and fire regime depend on plant functional traits. Journal of Ecology, 102(6), 1572–1581. 10.1111/1365-2745.12306

[ece38717-bib-0025] Enright, N. J. , & Thomas, I. (2008). Pre‐European fire regimes in Australian ecosystems. Geography Compass, 2(4), 979–1011. 10.1111/j.1749-8198.2008.00126.x

[ece38717-bib-0026] Fairman, T. A. , Nitschke, C. R. , & Bennett, L. T. (2015). Too much, too soon? A review of the effects of increasing wildfire frequency on tree mortality and regeneration in temperate eucalypt forests. International Journal of Wildland Fire, 25(8), 831–848. 10.1071/WF15010

[ece38717-bib-0027] Fernández, C. , Vega, J. A. , & Fonturbel, T. (2013). Does fire severity influence shrub resprouting after spring prescribed burning? Acta Oecologica, 48, 30–36. 10.1016/j.actao.2013.01.012

[ece38717-bib-0028] Flannigan, M. , Cantin, A. S. , De Groot, W. J. , Wotton, M. , Newbery, A. , & Gowman, L. M. (2013). Global wildland fire season severity in the 21st century. Forest Ecology and Management, 294, 54–61. 10.1016/j.foreco.2012.10.022

[ece38717-bib-0029] Foster, C. N. , Barton, P. S. , MacGregor, C. I. , Catford, J. A. , Blanchard, W. , & Lindenmayer, D. B. (2018). Effects of fire regime on plant species richness and composition differ among forest, woodland and heath vegetation. Applied Vegetation Science, 21, 132–143. 10.1111/avsc.12345

[ece38717-bib-0030] Fox, J. , & Weisbery, S. (2019). An R Companion to Applied Regression ‐ Google Books. Sage.

[ece38717-bib-0031] Gill, A. M. , & Moore, P. H. R. (1990). Fire intensities in Eucalyptus forests of southeastern Australia. In D. X. Viegas (Ed.), International Conference on Forest Fire Research, Coimbra, Portugal, B 24, 1–12.

[ece38717-bib-0032] Hammill, K. A. , & Bradstock, R. A. (2006). Remote sensing of fire severity in the Blue Mountains: Influence of vegetation type and inferring fire intensity. International Journal of Wildland Fire, 15(2), 213–226. 10.1071/WF05051

[ece38717-bib-0033] Hiers, Q. A. , Treadwell, M. L. , Dickinson, M. B. , Kavanagh, K. L. , Lodge, A. G. , Starns, H. D. , & Rogers, W. E. (2021). Grass bud responses to fire in a semiarid savanna system. Ecology and Evolution, 11(11), 6620–6633. 34141245 10.1002/ece3.7516 PMC8207346

[ece38717-bib-0034] Johnson, J. B. , & Omland, K. S. (2004). Model selection in ecology and evolution. Trends in Ecology & Evolution, 19(2), 101–108. 16701236 10.1016/j.tree.2003.10.013

[ece38717-bib-0035] Johnson, K. A. , Morrison, D. A. , & Goldsack, G. (1994). Postfire flowering patterns in *Blandfordia nobilis* (Liliaceae). Australian Journal of Botany, 42(1), 49–60. 10.1071/BT9940049

[ece38717-bib-0036] Keeley, J. E. (2009). Fire intensity, fire severity and burn severity: A brief review and suggested usage. International Journal of Wildland Fire, 18(1), 116. 10.1071/WF07049

[ece38717-bib-0037] Keeley, J. E. , & Brennan, T. J. (2012). Fire‐driven alien invasion in a fire‐adapted ecosystem. Oecologia, 169(4), 1043–1052. 10.1007/s00442-012-2253-8 22286083

[ece38717-bib-0038] Klimešová, J. , & Klimeš, L. (2007). Bud banks and their role in vegetative regeneration–A literature review and proposal for simple classification and assessment. Perspectives in Plant Ecology, Evolution and Systematics, 8(3), 115–129. 10.1016/j.ppees.2006.10.002

[ece38717-bib-0039] Knox, K. J. , & Clarke, P. J. (2011). Fire severity and nutrient availability do not constrain resprouting in forest shrubs. Plant Ecology, 212(12), 1967–1978. 10.1007/s11258-011-9956-5

[ece38717-bib-0040] Knox, K. J. , & Clarke, P. J. (2016). Measuring fire severity: Are canopy, understorey and below‐ground measures coupled in sclerophyll forest fires? Plant Ecology, 217(6), 607–615. 10.1007/s11258-016-0609-6

[ece38717-bib-0041] Lamont, B. B. , & Downes, K. S. (2011). Fire‐stimulated flowering among resprouters and geophytes in Australia and South Africa. Plant Ecology, 212(12), 2111–2125. 10.1007/s11258-011-9987-y

[ece38717-bib-0042] Le Breton, T. D. , Lyons, M. B. , Nolan, R. H. , Penman, T. , Williamson, G. J. , & Ooi, M. K. J. (2022). Megafire‐induced interval squeeze threatens vegetation at landscape scales. Frontiers in Ecology and the Environment. 10.1002/fee.2482

[ece38717-bib-0043] Lloret, F. , & López‐Soria, L. (1993). Resprouting of *Erica multiflora* after experimental fire treatments. Journal of Vegetation Science, 4(3), 367–374. 10.2307/3235595

[ece38717-bib-0044] Martínez‐Vilalta, J. , Sala, A. , Asensio, D. , Galiano, L. , Hoch, G. , Palacio, S. , Piper, F. I. , & Lloret, F. (2016). Dynamics of non‐structural carbohydrates in terrestrial plants: A global synthesis. Ecological Monographs, 86(4), 495–516. 10.1002/ecm.1231

[ece38717-bib-0045] McLauchlan, K. K. , Higuera, P. E. , Miesel, J. , Rogers, B. M. , Schweitzer, J. , Shuman, J. K. , Tepley, A. J. , Varner, J. M. , Veblen, T. T. , Adalsteinsson, S. A. , Balch, J. K. , Baker, P. , Batllori, E. , Bigio, E. , Brando, P. , Cattau, M. , Chipman, M. L. , Coen, J. , Crandall, R. , … Watts, A. C. (2020). Fire as a fundamental ecological process: Research advances and frontiers. Journal of Ecology, 108(5), 2047–2069. 10.1111/1365-2745.13403

[ece38717-bib-0046] McLoughlin, L. C. (1998). Season of burning in the Sydney region: The historical records compared with recent prescribed burning. Australian Journal of Ecology, 23(4), 393–404. 10.1111/j.1442-9993.1998.tb00744.x

[ece38717-bib-0047] Miller, R. G. , Tangney, R. , Enright, N. J. , Fontaine, J. B. , Merritt, D. J. , Ooi, M. K. J. , Ruthrof, K. X. , & Miller, B. P. (2019). Mechanisms of Fire Seasonality Effects on Plant Populations. Trends in Ecology & Evolution, 34(12), 1104–1117. 10.1016/j.tree.2019.07.009 31399287

[ece38717-bib-0048] Miller, R. G. , Tangney, R. , Enright, N. J. , Fontaine, J. B. , Merritt, D. J. , Ooi, M. K. J. , Ruthrof, K. X. , & Miller, B. P. (2020). Fire seasonality mechanisms are fundamental for understanding broader fire regime effects. Trends in Ecology & Evolution, 35(10), 869–871. 10.1016/j.tree.2020.08.002 32917394

[ece38717-bib-0049] Moreno, J. M. , & Oechel, W. C. (1989). A simple method for estimating fire intensity after a burn in California chaparral. Acta Oecologica, 10(1), 57–68.

[ece38717-bib-0050] Moreno, J. M. , & Oechel, W. C. (1991). Fire intensity and herbivory effects on postfire resprouting of *Adenostoma fasciculatum* in southern California chaparral. Oecologia, 85(3), 429–433. 10.1007/BF00320621 28312050

[ece38717-bib-0051] Nield, A. P. , Enright, N. J. , & Ladd, P. G. (2016). Fire‐stimulated reproduction in the resprouting, non‐serotinous conifer *Podocarpus drouynianus* (Podocarpaceae): The impact of a changing fire regime. Population Ecology, 58(1), 179–187. 10.1007/s10144-015-0509-y

[ece38717-bib-0052] Nolan, R. H. , Collins, L. , Leigh, A. , Ooi, M. K. J. , Curran, T. J. , Fairman, T. A. , Resco de Dios, V. , & Bradstock, R. (2021). Limits to post‐fire vegetation recovery under climate change. Plant, Cell & Environment, 44(11), 3471–3489. 10.1111/pce.14176 34453442

[ece38717-bib-0053] Ooi, M. K. (2019). The importance of fire season when managing threatened plant species: A long‐term case‐study of a rare *Leucopogon* species (Ericaceae*)* . Journal of Environmental Management, 236, 17–24. 10.1016/j.jenvman.2019.01.083 30711738

[ece38717-bib-0054] Ott, J. P. , Klimešová, J. , & Hartnett, D. C. (2019). The ecology and significance of below‐ground bud banks in plants. Annals of Botany, 123(7), 1099–1118. 10.1093/aob/mcz051 31167028 PMC6612937

[ece38717-bib-0055] Paroissien, R. , & Ooi, M. K. J. (2021). Effects of fire season on the reproductive success of the post‐fire flowerer *Doryanthes excelsa* . Environmental and Experimental Botany, 192, 104634. 10.1016/j.envexpbot.2021.104634

[ece38717-bib-0056] Pausas, J. G. (2019). Generalized fire response strategies in plants and animals. Oikos, 128, 147–153. 10.1111/oik.05907

[ece38717-bib-0057] Pausas, J. G. , & Keeley, J. E. (2014). Evolutionary ecology of resprouting and seeding in fire‐prone ecosystems. New Phytologist, 204(1), 55–65. 10.1111/nph.12921 25298997

[ece38717-bib-0058] Pausas, J. G. , & Keeley, J. E. (2017). Epicormic resprouting in fire‐prone ecosystems. Trends in Plant Science, 22(12), 1008–1015. 10.1016/j.tplants.2017.08.010 28927652

[ece38717-bib-0059] Pausas, J. G. , & Keeley, J. E. (2021). Wildfires and global change. Frontiers in Ecology and the Environment. 10.1002/fee.2359

[ece38717-bib-0060] Pérez, B. , & Moreno, J. M. (1998). Methods for quantifying fire severity in shrubland‐fires. Plant Ecology, 139(1), 91–101.

[ece38717-bib-0061] Pyke, G. H. (1982). Fruit set in *Lambertia formosa* Sm. (Proteaceae). Australian Journal of Botany, 30(1), 39–45. 10.1071/BT9820039

[ece38717-bib-0062] Pyke, G. H. (2017). Fire‐stimulated flowering: A review and look to the future. Critical Reviews in Plant Sciences, 36(3), 179–189. 10.1080/07352689.2017.1364209

[ece38717-bib-0063] RStudio Team . (2020). RStudio: Integrated Development for R. RStudio, PBC. http://www.rstudio.com/

[ece38717-bib-0064] Schutz, A. E. N. , Bond, W. J. , & Cramer, M. D. (2009). Juggling carbon: Allocation patterns of a dominant tree in a fire‐prone savanna. Oecologia, 160, 235–246. 10.1007/s00442-009-1293-1 19214583

[ece38717-bib-0065] Smith, M. G. , Arndt, S. K. , Miller, R. E. , Kasel, S. , & Bennett, L. T. (2018). Trees use more non‐structural carbohydrate reserves during epicormic than basal resprouting. Tree Physiology, 38, 1779–1791. 10.1093/treephys/tpy099 30219862

[ece38717-bib-0066] Strydom, T. , Kraaij, T. , Difford, M. , & Cowling, R. M. (2020). Fire severity effects on resprouting of subtropical dune thicket of the Cape Floristic Region. PeerJ, 8, e9240. 10.7717/peerj.9240 32566395 PMC7293192

[ece38717-bib-0067] Tangney, R. , Miller, R. G. , Enright, N. J. , Fontaine, J. B. , Merritt, D. J. , Ooi, M. K. J. , Ruthrof, K. X. , & Miller, B. P. (2020). Seed dormancy interacts with fire seasonality mechanisms. Trends in Ecology & Evolution, 35(12), 1057–1059. 10.1016/j.tree.2020.09.008 33121751

[ece38717-bib-0068] Taylor, J. E. , Monamy, V. , & Fox, B. J. (1998). Flowering of *Xanthorrhoea fulva*: The effect of fire and clipping. Australian Journal of Botany, 46(2), 241–251. 10.1071/BT96100

[ece38717-bib-0069] Thomsen, A. , & Ooi, M. (2021). Shifting season of fire and its impact on reproductive effort in resprouting plants. Figshare, Dataset. 10.6084/m9.figshare.15079248.v2 PMC8931712 35342578

[ece38717-bib-0070] Whelan, R. J. (1995). The ecology of fire. Cambridge University Press.

[ece38717-bib-0071] Whight, S. , & Bradstock, R. (1999). Indices of fire characteristics in sandstone heath near Sydney, Australia. International Journal of Wildland Fire, 9(2), 145–153. 10.1071/WF00012

[ece38717-bib-0072] Zirondi, H. L. , Ooi, M. K. , & Fidelis, A. (2021). Fire‐triggered flowering is the dominant post‐fire strategy in a tropical savanna. Journal of Vegetation Science, 32(2), e12995. 10.1111/jvs.12995

