# Supplementary material for: Shifting season of fire and its interaction with fire severity: Impacts on reproductive effort in resprouting plants
Source: Ecol Evol. 2022 Mar 18;12(3):e8717. doi: 10.1002/ece3.8717 (PMC8931712; doi:10.1002/ece3.8717)
Supplement: Supplementary file 1 — Appendix S1 [file ECE3-12-e8717-s003.docx]

Supplementary material

## **Appendix S1:** Mean severity scores across sites for each season 2016-2018, calculated using minimum tip diameter. No significant difference was found in severity between seasons.

|  | autumn | | spring | |
| --- | --- | --- | --- | --- |
| year | site | severity | site | severity |
| 2016 | Cabbage tree | 0.496 | Beaumont Rd | 0.929 |
| 2016 | Heath trail | 0.82 | Beaumont Rd2 | 0.96 |
| 2016 | Heath trail2 | 0.776 | Harvey Trig Duckholes | 1.051 |
| 2016 | Lockley Pt | 0.536 | Harvey Trig Duckholes2 | 0.822 |
| 2016 | Woronora Dam | 1.277 | The Ridge | 1.417 |
| **2016** | **Total mean** | **0.798** |  | **1.044** |
| 2017 | Jinga West | 1.058 | Bluff track | 0.596 |
| 2017 | Somerville Rd | 0.569 | Church camps | 1.127 |
| 2017 | Somerville Rd2 | 1.004 | Church camps2 | 1.127 |
| 2017 | Somerville Rd3 | 0.582 | Deep Bay | 0.776 |
| 2017 | Somerville Rd4 | 0.669 | Haigh Ave | 0.865 |
| 2017 |  |  | Mckell Ave | 1.134 |
| 2017 |  |  | Provest Creek | 0.839 |
| **2017** | **Total mean** | **0.773** |  | **0.894** |
| 2018 | Cawleys Rd | 0.996 | Belinda Cres | 0.944 |
| 2018 | Cawleys Rd2 | 1.043 | Burraneer | 0.894 |
| 2018 | Cowan trail | 0.847 | Carroll Creek | 0.401 |
| 2018 | Jessica Gardens | 0.733 | Cottage Point | 1.208 |
| 2018 | Sir Bertram Dr | 1.27 | Downes St | 1.038 |
| 2018 | Sir Bertram Dr | 1.579 | Mt Bass Firetrail | 2.685 |
| 2018 | Sir Bertram Dr3 | 1.48 |  |  |
| 2018 | Wattamolla | 1.3 |  |  |
| **2018** | **Total mean** | **1.133** |  | **1.021** |
