# Supplementary figures and images for: Shifting season of fire and its interaction with fire severity: Impacts on reproductive effort in resprouting plants
Source: Ecol Evol. 2022 Mar 18;12(3):e8717. doi: 10.1002/ece3.8717 (PMC8931712; doi:10.1002/ece3.8717)

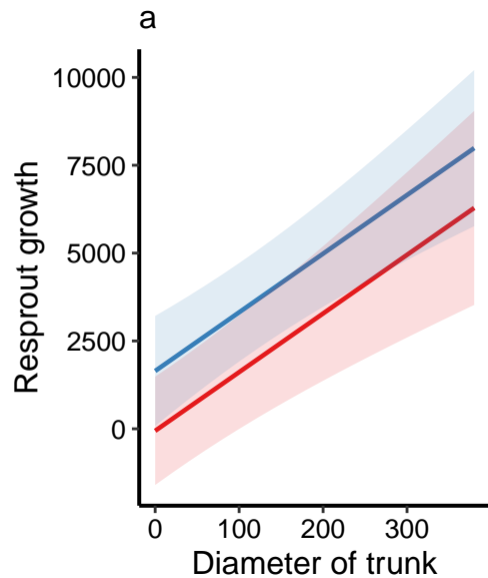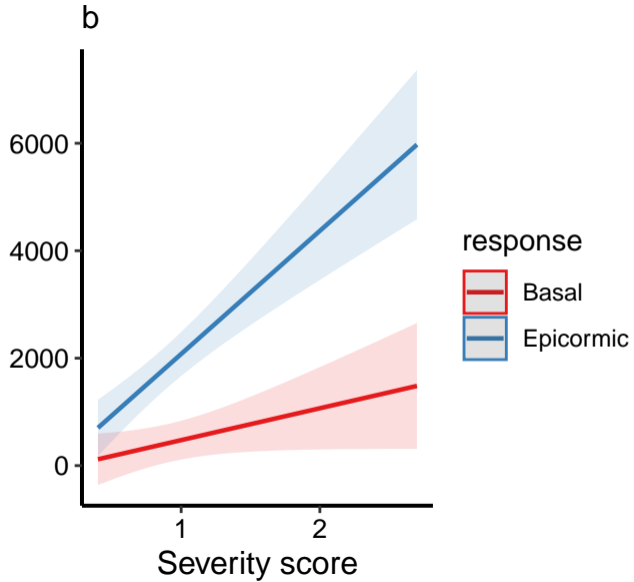

Supplement: Supplementary file 2 — Fig S2 [file ECE3-12-e8717-s001.pdf]
