# Supplementary material for: Shifting season of fire and its interaction with fire severity: Impacts on reproductive effort in resprouting plants
Source: Ecol Evol. 2022 Mar 18;12(3):e8717. doi: 10.1002/ece3.8717 (PMC8931712; doi:10.1002/ece3.8717)
Supplement: Supplementary file 3 — Appendix S2 [file ECE3-12-e8717-s002.docx]

Supplementary material 2

**Appendix S2:** Best supported models for resprouting vigour in a) *Banksia serrata* and b) *Leptospermum trinervium* at the species level. Predicted resprouting vigour was best supported by the full model for both species including resprout response x severity, age, diameter, season and site as a random factor. Only diameter was a significant factor for *B. serrata* (DF=1, ꭓ2=26.158, P<0.0001). Influencing factors for *L. trinervium* included resprout response x severity (DF=1, ꭓ2=13.17, P=0.0003), age of resprouts (DF=1, ꭓ2=13.733, P=0.0003), and diameter (DF=1, ꭓ2=8.49, P=0.0039). Figures depict resprouting vigour for both individuals that resprouted basally and epicormically with a) changing diameter in *B. serrata* and b) increasing fire severity in *L. trinervium.*
